# Analysis of Specific IgG4 Antibody and sIgG4/sIgE Antibody Ratio as Marker of Immune Tolerance to IgE-Mediated Response in Allergic Children

**DOI:** 10.3390/children12121679

**Published:** 2025-12-10

**Authors:** HyeonA Kim, Ben Kang, Bong Seok Choi

**Affiliations:** Department of Pediatrics, School of Medicine, Kyungpook National University, Daegu 41944, Republic of Korea; crystalha50@knuh.kr (H.K.);

**Keywords:** immunoglobulin G4, immunoglobulin E, child, immune tolerance, allergen

## Abstract

Background: The diagnosis and treatment of allergic diseases remain challenging because the results of allergy testing do not always correlate with clinical manifestations. For instance, although a specific IgE (sIgE) test for a particular allergen may be positive, some patients remain asymptomatic due to the development of immune tolerance. Recently, specific IgG4 (sIgG4) has been suggested as a potential indicator of immune tolerance. However, data in children are limited. Therefore, this study aimed to evaluate the clinical usefulness of sIgG4 testing and its potential role in assessing immune tolerance in pediatric allergic disease by measuring sIgE and sIgG4 levels. Methods: A total of 415 children with suspected allergic diseases were enrolled. All subjects underwent sIgE and sIgG4 testing for casein, egg white, peanut, *Dermatophagoides farinae* (*D. farinae*), dog dander, and birch. sIgE positivity was defined as a level of 0.35 kU/L or higher for each allergen. Test results and clinical characteristics were reviewed, and the association between symptom occurrence during actual allergen exposure and sIgG4 level or sIgG4/sIgE ratio was analyzed. Results: Among the 415 children, 253 were male (61%) and 162 were female (39%), with a median age of 5.77 years (IQR, 2.67–9.15). For inhalant allergens, sIgG4 levels did not differ between symptomatic and asymptomatic patients; however, the sIgG4/sIgE ratio was significantly higher in asymptomatic patients (*D. farinae*: *p* < 0.001, dog dander: *p* < 0.001, birch: *p* = 0.003)). For food allergens, significant differences in sIgE, sIgG4, and sIgG4/sIgE ratio were observed for casein (sIgE: *p* < 0.001, sIgG4: *p* < 0.001, sIgG4/sIgE: *p* < 0.001) and egg white (sIgE: *p* < 0.001, sIgG4: *p* < 0.001, sIgG4/sIgE: *p* < 0.001), while no significant associations were found for peanut. Conclusions: The correlation between sIgG4 and clinical symptom occurrence varied depending on the allergen. Although sIgG4 alone appears to have limited clinical value for inhalant allergens, the sIgG4/sIgE ratio may serve as a useful indicator of immune tolerance. For casein and egg white, both sIgG4 and the sIgG4/sIgE ratio demonstrated potential clinical utility for identifying immune tolerance; however, further investigation is needed to validate the role of sIgG4-based indicators in pediatric allergy management.

## 1. Introduction

Allergic diseases, including bronchial asthma, allergic rhinitis, food allergy, and atopic dermatitis, are prevalent in children [1]. These allergic diseases tend to appear sequentially over time, a phenomenon known as the allergic march. The allergic march refers to a series of events that begin with food allergies and/or atopic dermatitis in infancy and may gradually progress to respiratory diseases such as allergic rhinitis and bronchial asthma during the child’s growth. This process is clinically significant because allergic diseases develop in a correlated manner as children grow. Serum specific IgE (sIgE) testing and skin prick testing are widely used to identify causative allergens in these patients [2,3,4]. However, in clinical practice, cases are frequently encountered in which patients with positive sIgE results remain asymptomatic upon natural exposure to the corresponding allergen [5,6]. This discrepancy complicates the determination of clinically relevant allergens and may lead to unnecessary dietary or environmental restriction based solely on sensitization detected by testing [7,8]. Provocation testing is the gold standard for identifying symptomatic allergies, assessing tolerance, and determining the need for continued avoidance, and is particularly crucial for the diagnosis of food allergies [9,10,11,12]. Yet, these procedures are difficult to perform routinely, carry the risk of severe systemic reactions such as anaphylaxis. They are high-risk procedures and in many cases do not justify the benefit-risk ratio. Therefore, alternative diagnostic indicators are needed to better distinguish clinically significant allergies, reduce unnecessary provocation testing, and identify allergens that are tolerated through immune regulation [12,13].

Recently, antigen-specific IgG4 (sIgG4) has been investigated in IgE-mediated allergic diseases. sIgG4 is considered an immunologic marker associated with the mechanisms of allergen-specific immunotherapy, particularly in relation to the induction of immune tolerance during prolonged antigen exposure. This antibody subclass has been suggested to function as a blocking antibody that may interfere with IgE-mediated activation, indicating a potential role as a marker of immune tolerance [12,14,15,16,17].

However, limited data exist on the clinical significance of sIgG4 in pediatric populations. Therefore, this study aimed to measure sIgE and sIgG4 to various allergens in children and evaluate the usefulness of sIgG4, and the sIgG4/sIgE ratio, as indicators of immune tolerance and their potential applicability in clinical practice.

## 2. Materials and Methods

### 2.1. Subjects

From March 2019 to December 2021, a total of 415 children who visited Kyungpook National University Children’s Hospital underwent sIgE and sIgG4 testing for suspected allergic diseases. Children with underlying chronic diseases or those receiving medications were excluded. The study was approved by the Institutional Review Board (IRB) of Chilgok Kyungpook National University Hospital and was performed in accordance with relevant ethical guidelines (IRB number: 2021-05-006).

### 2.2. Methods

A retrospective review of medical records was performed to obtain demographic and clinical information, including sex, age at the time of testing, and diagnosed allergic diseases.

#### 2.2.1. Laboratory Tests

Specific IgE antibody testing was performed using the CAP radioallergosorbent technique (UniCAP, Pharmacia, Uppsala, Sweden) to identify sensitization to inhalant and food allergens. Inhalant allergens included *D. farinae*, dog dander, and birch, and food allergens included egg white, casein, and peanut. A level of 0.35 kU/L or higher was defined as positive [18,19].

Specific IgG4 antibodies were tested using the ImmunoCAP fluoroenzymeimmunoassay (UniCAP, Phadia AB, Uppsala, Sweden) for the same six allergens. Additional laboratory parameters, including total eosinophil count, total serum IgE, and eosinophilic cationic protein (ECP), were also measured.

#### 2.2.2. Classification of Patient Groups by Symptom Presence

Patients with an sIgE level of 0.35 kU/L or higher for a given allergen were subdivided into symptomatic and asymptomatic groups based on the presence of clinically relevant symptoms upon actual exposure to that allergen. Symptom presence or absence was confirmed through structured interviews with patients and caregivers. Repeated respiratory symptoms of rhinitis (sneezing, nasal congestion, runny nose) or asthma (cough, wheezing, shortness of breath) during exposure to an inhalant allergen, or allergic reactions such as skin rash, urticaria, angioedema, or anaphylaxis, were considered evidence of clinical reactivity. For food allergens, patients were classified as symptomatic if they repeatedly developed allergic reactions within 2 h of ingesting the raw or unprocessed form of the food [20]. Current dietary treatment, such as avoidance therapy, was not considered.

Symptomatic criteria were further defined separately for each inhalant allergen.

For *D. farinae*, the symptomatic group included patients who experienced rhinorrhea, nasal congestion, sneezing, eye redness, or rash in dust-rich environments, without significant seasonal variation, and whose symptoms improved with mite-specific environmental control.

For birch, symptomatic status was defined as recurrent allergic symptoms during the spring season when pollen concentrations are high. For dog dander, symptomatic patients were defined as those who exhibited allergic reactions within 2 h of direct exposure to dogs.

#### 2.2.3. Study Design

Patients were classified into groups based on the presence or absence of clinical reaction symptoms upon actual allergen exposure, along with their IgE levels. Associations among sIgE level, sIgG4 level, and the sIgG4/sIgE ratio with symptom occurrence were then analyzed. Receiver operating characteristic (ROC) analysis was used to assess the diagnostic utility of sIgE, sIgG4, and the sIgG4/sIgE ratio for each allergen. The area under the curve (AUC) was calculated, and optimal cutoff values were determined using the Youden index to identify sensitivity, specificity, positive predictive value, negative predictive value, and cutoff levels with 95% confidence.

### 2.3. Statistical Method

For statistical comparison between groups, a χ2 test or Fisher’s exact test was used for categorical variables, and a Student’s *t*-test or Wilcoxon rank-sum test was used for continuous variables. Comparative data for continuous variables are reported as median with interquartile range (IQR) or mean with standard deviation. Receiver operating characteristic (ROC) curve analysis was conducted to investigate the performance of the ImmunoCAP test. Differences were considered statistically significant for *p* values < 0.05. All statistical analyses were performed using R version 4.3.1 (http://www.r-project.org (accessed on 3 December 2025)).

## 3. Results

### 3.1. Demographics and Clinical Characteristics of the Subjects

Among the 415 enrolled patients, the median age of the participants was 5.77 years (IQR, 2.67–9.15). and 253 (61%) were boys and 162 (39%) were girls, yielding a male-to-female ratio of 1.56:1. Chronic urticaria was the most common allergic diagnosis, accounting for 79 patients (19.0%), followed by asthma in 70 (16.9%) and allergic rhinitis in 64 (15.4%) (Table 1).

Egg white-specific sIgE was positive in 153 patients; however, only 49 (32.0%) exhibited clinical symptoms following ingestion. Symptomatic responses to peanut and casein were identified in 24.8% and 24.6% of patients with positive sIgE results, respectively. Among inhalant allergens, *D. farinae* showed the highest rate of symptomatic presentation (64.3%), followed by birch (62.6%) and dog dander (37.5%) in patients with positive sIgE results (Table 2). The median total serum IgE level was 91.8 IU/mL (IQR, 27.0–295.9), and elevated levels of 200 IU/mL or higher were observed in 138 patients (33.3%). The median peripheral eosinophil count was 260 cells/mm^3^ (IQR, 130–480), and eosinophilia (≥500/mm^3^) was present in 101 patients (24.3%) (Table 2).

### 3.2. Association of Specific Antibodies (sIgE, sIgG4, and sIgG4/sIgE Ratio) with Clinical Symptoms by Allergen

For inhalant allergens, sIgG4 levels did not differ significantly between symptomatic and asymptomatic patients. In contrast, sIgE levels were significantly higher in symptomatic patients for all three inhalant allergens (*D. farinae*: *p* < 0.003, dog dander: *p* < 0.001, birch: *p* < 0.001). Additionally, the sIgG4/sIgE ratio was significantly elevated in asymptomatic patients sensitized to these allergens (*D. farinae*: *p* < 0.001, dog dander: *p* < 0.001, birch: *p* = 0.003). For food allergens, for casein (sIgE: *p* < 0.001, sIgG4: *p* < 0.001, sIgG4/sIgE ratio: *p* < 0.001) and egg white (sIgE: *p* < 0.001, sIgG4: *p* < 0.001, sIgG4/sIgE ratio: *p* < 0.001), sIgG4 and sIgG4/E ratio were significantly higher in the asymptomatic group, while sIgE was higher in the symptomatic group. In other words, significant differences were observed between the symptomatic and asymptomatic groups for all three antibody parameters. In peanut-sensitized patients, only sIgE differed significantly, while sIgG4 and the sIgG4/sIgE ratio did not show significant distinctions between symptomatic and asymptomatic groups (Table 3).

### 3.3. ROC Curve of Specific Antibodies According to Allergen

#### 3.3.1. Inhalant Allergens

**Figure 1:**
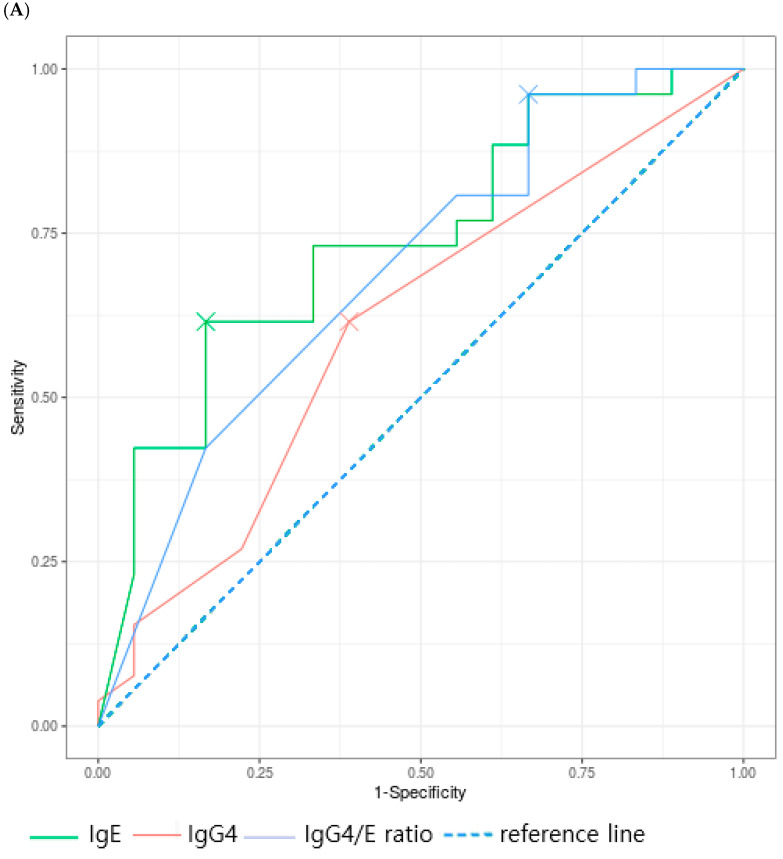

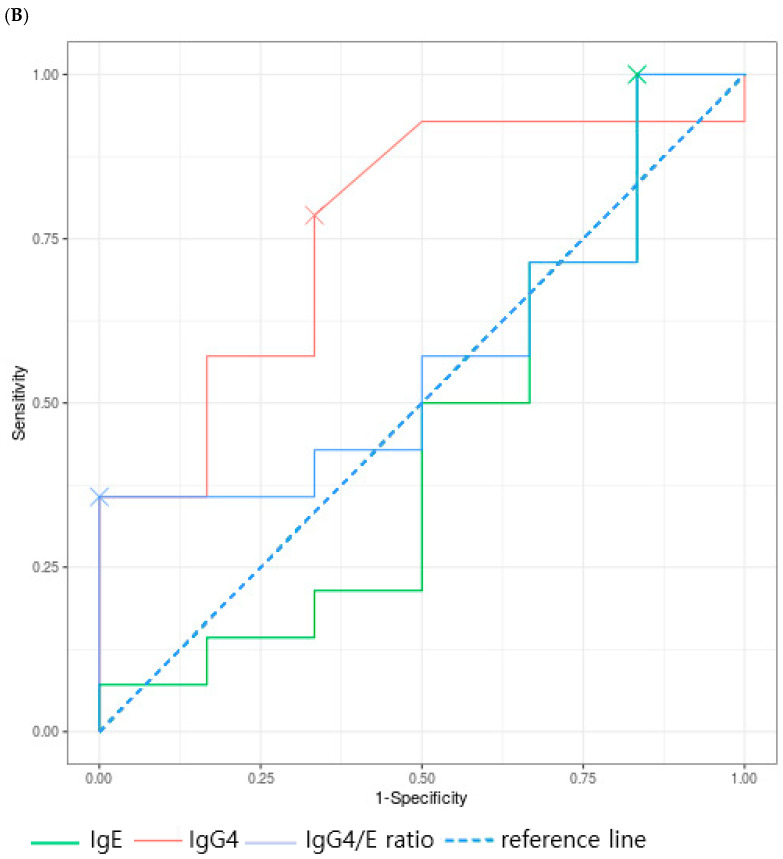

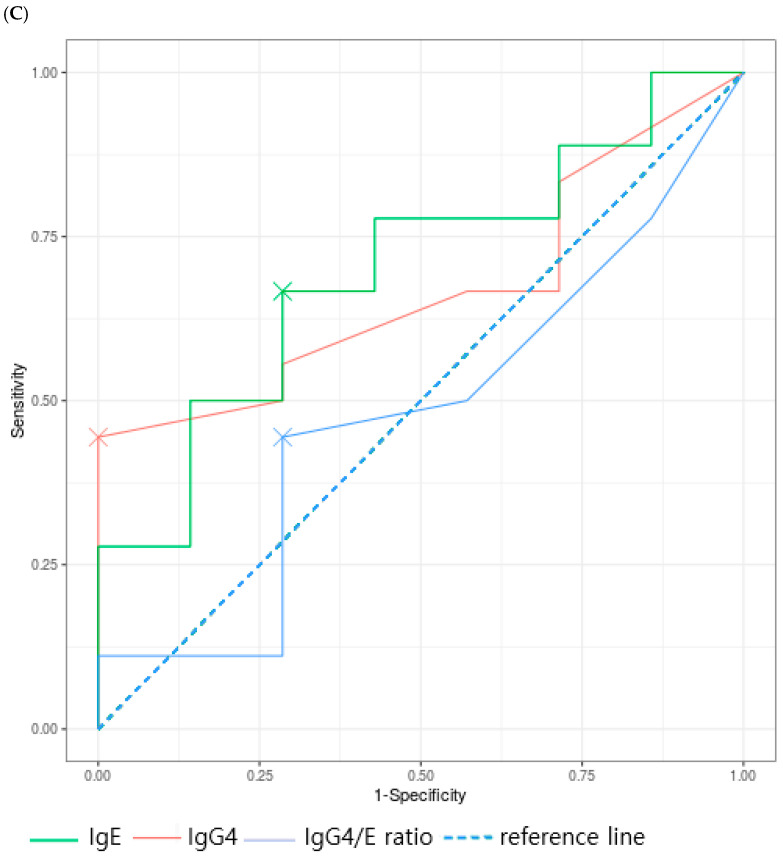
Receiver operating characteristic curve of inhalant allergen (**A**) *Dermatophagoides farinae*, (**B**) Dog dander, (**C**) Birch.

For sIgG4, dog dander demonstrated an AUC of 0.762 (*p* < 0.001), whereas sIgG4 for the other inhalant allergens did not exhibit meaningful diagnostic performance. For the sIgG4/sIgE ratio, a significant AUC was identified only for *D. farinae* (AUC 0.691, *p* = 0.009), with no other inhalant allergens demonstrating diagnostic relevance (Figure 1A–C). Optimal cutoff values were calculated for each allergen. In patients sensitized to dog dander, the cutoff values were 0.6 kU/L for sIgE (sensitivity 100%, specificity 16.7%) and 3.8 kU/L for sIgG4 (sensitivity 78.6%, specificity 66.7%). For *D. farinae*, the optimal cutoff values were 27.7 kU/L for sIgE (sensitivity 61.5%, specificity 83.3%) and 0.06 for the sIgG4/sIgE ratio (sensitivity 96.2%, specificity 33.3%). For birch, the sIgE cutoff value was 31 kU/L (sensitivity 66.7%, specificity 71.4%) and the sIgG4/sIgE ratio cutoff was 0.03 (sensitivity 44.4%, specificity 71.4%). The optimal cutoff values for sIgE were relatively high for both D. farinae and birch. In *D. farinae*, sIgE demonstrated high specificity and positive predictive value (83.3% and 84.2%, respectively), whereas the sIgG4/sIgE ratio demonstrated high sensitivity and negative predictive value (96.2% and 85.7%, respectively). In contrast, for dog dander, sIgE demonstrated the highest sensitivity and negative predictive value (100% for both), while the sIgG4/sIgE ratio demonstrated the highest specificity and positive predictive value (100% for both) (Table 4).

#### 3.3.2. Food Allergens

**Figure 2:**
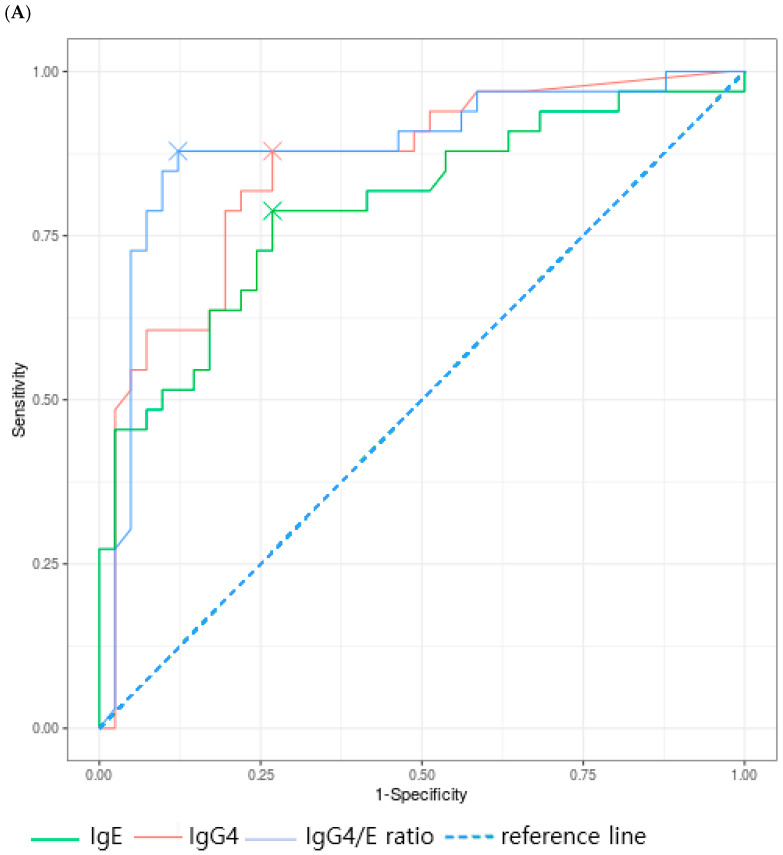

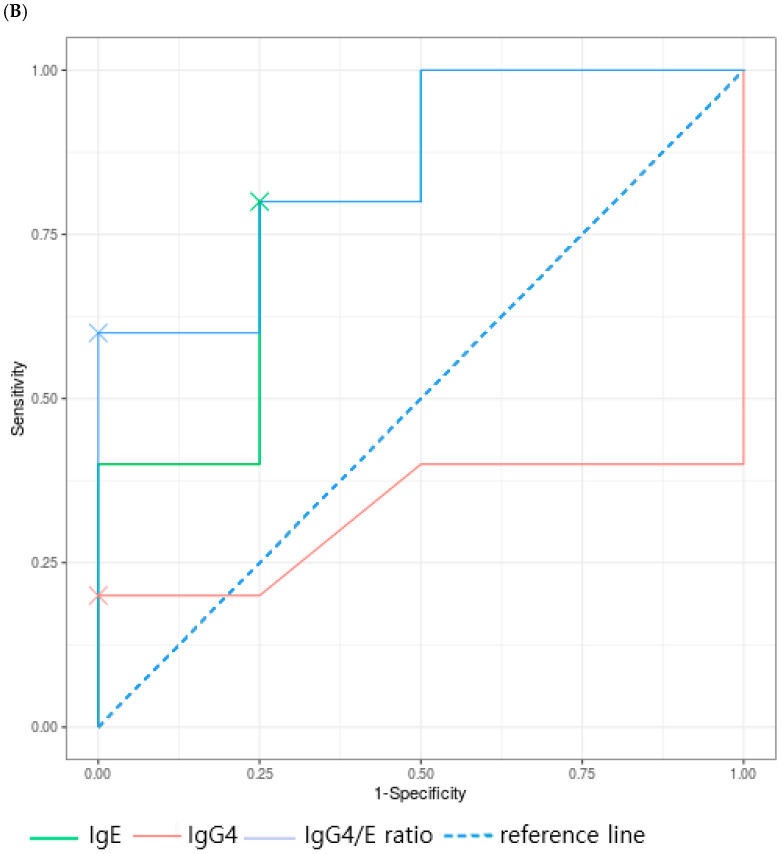

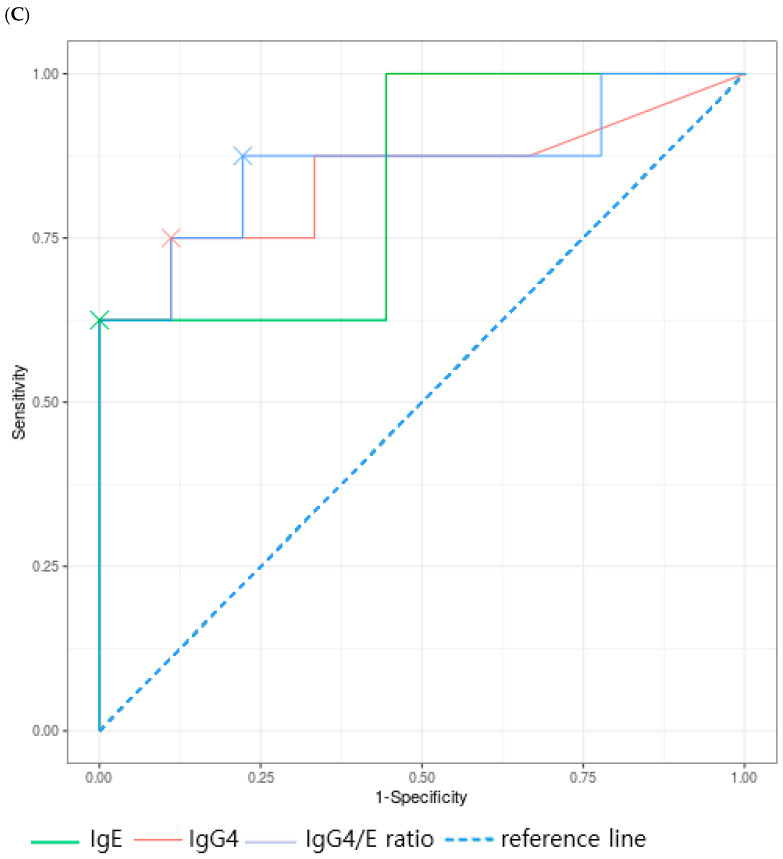
Receiver operating characteristic curve of food allergen (**A**) Egg white, (**B**) Peanut, (**C**) Casein.

ROC analysis revealed that sIgE, sIgG4, and the sIgG4/sIgE ratio showed significant diagnostic value in egg white and casein but not in peanut sensitization. For egg white, the AUC values were 0.793 for sIgE (*p* < 0.001), 0.851 for sIgG4 (*p* < 0.001), and 0.882 for the sIgG4/sIgE ratio (*p* < 0.001). For casein, AUC values were 0.833 for sIgE (*p* < 0.001), 0.840 for sIgG4 (*p* < 0.001), and 0.861 for the sIgG4/sIgE ratio (*p* < 0.001) (Figure 2A–C).

Optimal cutoff values for egg white were 1.45 kU/L for sIgE (sensitivity 78.8%, specificity 73.2%), 15.2 kU/L for sIgG4 (sensitivity 87.9%, specificity 73.2%), and 4.1 for the sIgG4/sIgE ratio (sensitivity 87.9%, specificity 87.8%). The sIgG4/sIgE ratio showed the best diagnostic performance, with a positive predictive value and negative predictive value of 85.3% and 90.0%, respectively. For casein, the optimal cutoff values were 6.47 kU/L for sIgE (sensitivity 62.5%, specificity 100%), 2.0 kU/L for sIgG4 (sensitivity 75.0%, specificity 88.9%), and 4.34 for the sIgG4/sIgE ratio (sensitivity 87.5%, specificity 77.8%). The sIgG4/sIgE ratio showed the highest sensitivity and negative predictive value (87.5% and 87.5%), whereas sIgE alone demonstrated the highest specificity and positive predictive value (100% for both). For peanut, sIgE showed a significant AUC of 0.800 (*p* = 0.002), with relatively high sensitivity, specificity, and predictive values. However, sIgG4 and the sIgG4/sIgE ratio showed high specificity and positive predictive value (100% for both), but low sensitivity and negative predictive value, limiting their clinical diagnostic usefulness (Table 5).

## 4. Discussion

In this study, sIgG4 and the sIgG4/sIgE ratio for casein and egg white demonstrated a meaningful correlation with the presence of clinical symptoms during exposure to the causative food allergens. However, the degree of correlation varied depending on the allergen. sIgG4 levels did not correlate with symptom occurrence for inhalant allergens or peanut. In contrast, the sIgG4/sIgE ratio showed a significant association with clinical responses to casein, egg white, *D. farinae*, dog dander, and birch, with the exception of peanut. Analysis of the sIgG4/sIgE ratio is generally found to be higher in patients who are asymptomatic but show positive allergen test results than in those who are symptomatic and test positive.

An increase in sIgG4 has been observed following continuous allergen exposure [18,21,22,23] or during allergen-specific immunotherapy [24], and this rise is believed to be associated with the development of immune tolerance. Several immunological mechanisms have been proposed to explain this relationship. Exposure to allergens stimulates the secretion of interleukin-10 (IL-10), which suppresses mast cell and eosinophil activation, and modulates Th1 and Th2 cell function. These effects induce cellular immune tolerance, inhibit IgE production, and promote IgG4 production [25]. In support of this, healthy beekeepers without allergic disease demonstrate activation of IL-10–secreting regulatory T cells. Furthermore, sIgG4 has been described as a blocking antibody capable of competing with IgE for allergen binding on effector cells such as mast cells and neutrophils, thereby reducing IgE-mediated cellular activation [24].

Findings from previous studies on sIgG4 in inhalant allergies are consistent with our results. Burnett et al. [19] reported that, in cat allergy, symptomatic individuals exhibited higher cat-sIgE and sIgG4 levels but a lower sIgG4/sIgE ratio compared with asymptomatic individuals. A similar pattern was observed for dog allergy, where dog-sIgE was higher and the sIgG4/sIgE ratio was lower in symptomatic subjects, although sIgG4 levels themselves did not differ between groups. Likewise, the present study showed that inhalant allergen sIgG4 alone is insufficient for predicting symptom development. The higher sIgG4/sIgE ratio in asymptomatic patients suggests a possible role in immune tolerance, but its standalone diagnostic value appears limited.

For food allergens, previous studies have yielded heterogeneous results regarding the ability of sIgG4 to indicate immune tolerance [11,26]. Food allergies, particularly peanut allergy, can lead to severe systemic reactions, and even minimal accidental exposure may result in life-threatening events [16]. The potential role of sIgG4 as a blocking antibody is supported by observations that elevated milk-specific sIgG4 levels are associated with tolerance in individuals who remain asymptomatic despite sensitization [27]. Conversely, other studies indicate that increased food-specific sIgG4 may also be present in healthy, nonallergic adults and children, suggesting that elevated sIgG4 levels may simply reflect repeated dietary exposure rather than immune tolerance, thus limiting its reliability as a diagnostic marker [11].

In this study, we investigated the clinical relevance of sIgG4 in three food allergens. Previous research has shown that casein-specific IgE is a better predictor of milk allergy than whole-milk sIgE [20,28], which is why casein was selected as the milk allergen in this analysis. Among patients who were casein-sIgE-positive, statistically significant differences in sIgE, sIgG4, and the sIgG4/sIgE ratio were observed between symptomatic and asymptomatic groups, consistent with prior findings [20]. ROC analysis confirmed the diagnostic utility of these specific antibody measurements, with AUC values of 0.833 for casein-sIgE, 0.840 for sIgG4, and 0.861 for the sIgG4/sIgE ratio. The optimal cutoff values were 6.47 kU/L for sIgE, 2.00 kU/L for sIgG4, and 4.34 for the sIgG4/sIgE ratio. The positive and negative predictive values were relatively high (sIgE: 100% and 75.0%, sIgG4: 85.7% and 80.0%, sIgG4/sIgE ratio: 77.8% and 87.5%). One Korean study reported cutoff values for milk-sIgE, casein-sIgE, and the casein-sIgG/sIgE ratio of 0.73 kU/L, 0.14 kU/L, and 36.9 in children younger than 1 year, and 1.34 kU/L, 1.91 kU/L, and 13.7 in those older than 1 year [20]. The differences between these previous reports and the present results likely reflect variations in study population characteristics and sample size. Similarly, for egg white, significant differences were identified in sIgE, sIgG4, and sIgG4/sIgE ratios between symptomatic and asymptomatic groups among children with positive egg white-sIgE. The AUC values were 0.793 for sIgE, 0.851 for sIgG4, and 0.882 for the sIgG4/sIgE ratio. The optimal cutoff values were 1.45 kU/L for sIgE, 15.2 kU/L for sIgG4, and 4.1 for the sIgG4/sIgE ratio. Although predictive values were high for all three antibody markers, sIgG4/sIgE demonstrated the strongest diagnostic performance. Caubet et al. [15], found that, in egg-allergic children, higher ratios of ovalbumin- or ovomucoid-specific sIgE to sIgG4 were associated with a greater likelihood of symptom development, and a model incorporating both markers provided high predictive accuracy. They also suggested that interactions between sIgE and sIgG4 might improve identification of natural tolerance, with a cutoff egg white-sIgE level of 7 kU/L proposed as a predictor of tolerance development. Other studies have reported that ovalbumin-specific sIgG4 alone can serve as an independent predictor of tolerance to uncooked eggs, and that the ovalbumin-sIgE/sIgG4 ratio has superior prognostic value over sIgE alone in determining allergy persistence or resolution [29]. In our study, egg white-specific assays were used rather than component-resolved diagnostics, which may limit direct comparison with these reports. However, one study demonstrated that the egg white sIgE/sIgG4 ratio significantly predicted oral food challenge (OFC) outcomes, with an AUC of 0.847, outperforming IgE (0.697) and IgG4 (0.724) alone, and achieving a negative predictive value of 90% for identifying children unlikely to react during controlled food exposure [30]. These findings reinforce the clinical value of incorporating the IgE/IgG4 ratio into diagnostic strategies for food allergy. Continued research is warranted to validate the potential of sIgG4-based indicators in predicting immune tolerance, particularly in egg allergy.

For peanut allergy, previous studies have produced conflicting results regarding the utility of sIgG4 as a marker of immune tolerance. One study suggested that sIgG4 measurement may help differentiate symptomatic from asymptomatic patients [31], whereas others have reported that sIgG4 does not contribute meaningfully to distinguishing between these groups [32,33], In the present study, only sIgE differed significantly between symptomatic and asymptomatic individuals sensitized to peanut, and ROC curve analysis did not support a diagnostic role for sIgG4 or the sIgG4/sIgE ratio. These findings are consistent with earlier studies indicating limited association between peanut-specific antibody profiles and clinical reactivity [34].

Variation in the performance of sIgG4 across food allergens was also observed. One study reported that IgG4 levels were markedly higher in cow’s milk compared with peanut among food allergens [31,35], which may explain why sIgG4 correlated with symptom status for casein but not for peanut in our study. Importantly, although these data are not included in the main result tables, our analysis confirmed that the probability of symptomatic response decreased significantly with increasing sIgG4 levels for both casein (odds ratio 0.88, *p* = 0.006) and egg white (odds ratio 0.91, *p* = 0.007). These findings suggest that sIgG4 might contribute to tolerance-associated mechanisms, even if its clinical measurement alone is not sufficient for peanut allergy determination.

This study has several limitations. Notably, the absence of direct confirmation of allergic reactions through provocation testing may affect the diagnostic accuracy and reliability of our findings. Since symptom presence, particularly for inhalant allergens such as *D. farinae* and birch, was primarily determined by caregiver or patient reporting, there is an inherent risk of subjective bias. Future studies incorporating standardized allergen provocation tests will enable more objective and reliable diagnoses. Additionally, this study was conducted at a single institution with a limited sample size, which may affect the generalizability of the results.

Because this study has these limitations, future research incorporating provocation testing as well as multicenter, large-scale studies to enhance diagnostic accuracy and research reliability would result in more robust findings. In summary, this study evaluated the diagnostic value of sIgG4 and the sIgG4/sIgE ratio as potential indicators of immune tolerance and explored optimal cutoff thresholds for clinical application. The correlation between sIgG4 and clinical symptoms varied depending on the specific allergen. Both sIgG4 and the sIgG4/sIgE ratio demonstrated significant value in casein and egg white sensitization as markers of immune tolerance. In fact, considering that most food allergies in children are to milk and eggs, it is hoped that additional research on these most problematic antigens will serve as a tool to confirm immune tolerance and reduce unnecessary provocation tests in clinical practice. However, further research with larger, multicenter cohorts and standardized clinical challenge testing is needed to fully establish the clinical utility of these measurements.

## Figures and Tables

**Table 1:** Demographic and clinical characteristics of the study population (*n* = 415).

Characteristics	Result
Age (years)	5.77 (2.67; 9.15)
Male:Female	253:162 (1.56:1)
Diagnosed allergic diseases *n* (%)	
Asthma	70 (16.9%)
Allergic rhinitis	64 (15.4%)
Atopic dermatitis	63 (15.2%)
Chronic urticaria	79 (19.0%)
Food allergy	44(10.6%)
Other (e.g., viral rash, allergic conjunctivitis, chronic cough, anaphylaxis)	95(22.9%)

**Table 2:** Laboratory analysis of the subjects (*n* = 415).

Variable	Results	No. (%)
Presence of symptoms among patients with allergen-specific IgE positivity		
*Dermatophagoides farina*	171:110 (64.3%)	
Dog dander	96:36 (37.5%)	
Birch	107:67 (62.6%)	
Egg white	153:49 (32.0%)	
Peanut	105:26 (24.8%)	
Casein	65:16 (24.6%)	
Total eosinophil count (/mm^3^)	260 (130; 480)	
Eosinophilia (>500) *n* (%)		101 (24.3%)
Total serum IgE (IU/mL)	91.8 (27.0; 295.9)	
Elevated IgE (>200) *n* (%)		138 (33.3%)
ECP (µg/L)	17.9 (9.7; 34.1)	

**Table 3:** Comparison of antibody levels between symptomatic and asymptomatic groups according to allergen.

	Symptomatic	Asymptomatic	*p* Value
*Dermatophagoides farinae* (N)	110	61	
sIgG4	0.20 (0.10; 0.30)	0.10 (0.10; 0.30)	0.247
sIgE	28.30 (8.62; 72.50)	5.87 (0.91; 27.00)	<0.003
sIgG4/E ratio	0.01 (0.00; 0.02)	0.03 (0.01; 0.15)	<0.001
Dog dander (N)	36	60	
sIgG4	2.25 (1.25; 4.20)	2.15 (0.80; 4.55)	0.883
sIgE	4.75 (1.36; 21.4)	0.86 (0.49; 2.38)	<0.001
sIgG4/E ratio	0.40 (0.07; 1.21)	1.62(0.39; 5.10)	<0.001
Birch (N)	67	40	
sIgG4	0.40 (0.10; 1.30)	0.40 (0.15; 1.20)	0.951
sIgE	25.00 (2.29; 66.85)	3.98 (0.77; 15.85)	<0.001
sIgG4/E ratio	0.03 (0.01; 0.10)	0.15 (0.02; 0.47)	0.003
Casein (N)	16	49	
sIgG4	0.90 (0.35; 3.70)	18.10 (7.90; 30.00)	<0.001
sIgE	4.60 (1.10; 21.15)	0.63 (0.45; 1.42)	<0.001
sIgG4/E ratio	0.12 (0.02; 1.48)	21.39 (7.38; 40.54)	<0.001
Egg white (N)	49	104	
sIgG4	0.50 (0.10; 6.60)	22.55 (12.00; 30.00)	<0.001
sIgE	5.13 (1.60; 16.50)	0.91 (0.58; 1.56)	<0.001
sIgG4/E ratio	0.16 (0.02; 0.63)	21.93 (7.71; 37.56)	<0.001
Peanut (N)	26	79	
sIgG4	0.30 (0.10; 1.00)	0.50 (0.10; 1.00)	0.371
sIgE	6.00 (0.95; 9.68)	1.41 (0.82; 3.79)	0.029
sIgG4/E ratio	0.08 (0.02; 0.17)	0.26 (0.05; 0.79)	0.019

**Table 4:** Diagnostic performance of inhalant allergen-specific antibodies.

Variable	Specific IgE (kU/L)			Specific IgG4 (kU/L)			Specific IgG4/E Ratio		
	*D. farinae*	Dog Dander	Birch	*D. farinae*	Dog Dander	Birch	*D. farinae*	Dog Dander	Birch
Area under curve	0.735	0.44	0.698	0.606	0.762	0.667	0.691	0.571	0.476
95% CI	0.584–0.886	0.237–0.882	0.47–0.927	0.444–0.768	0.521–1	0.451–0.882	0.536–0.846	0.295–0.848	0.266–0.782
*p* value	<0.001	<0.001	<0.001	0.2	<0.001	0.564	0.009	0.628	0.006
Cut off value	27.7	0.6	31	0.2	3.8	0.8	0.06	0.04	0.03
Sensitivity (%)	61.5	100	66.7	61.5	78.6	44.4	96.2	35.7	44.4
Specificity (%)	83.3	16.7	71.4	61.1	66.7	100	33.3	100	71.4
PPV (%)	84.2	73.7	85.7	69.6	84.6	100	67.6	100	80.0
NPV (%)	60.0	100	45.5	52.4	57.1	41.2	85.7	40	33.3

PPV, positive predictive value; NPV, negative predictive value.

**Table 5:** Diagnostic performance of food allergen-specific antibodies.

Variable	Specific IgE (kU/L)			Specific IgG4(kU/L)			Specific IgG4/E Ratio		
	Egg White	Peanut	Casein	Egg White	Peanut	Casein	Egg White	Peanut	Casein
Area under curve	0.793	0.8	0.833	0.851	0.25	0.84	0.882	0.85	0.861
95% CI	0.687–0.9	0.468–1	0.623–1	0.761–0.941	0.257–1	0.623–1	0.795–0.97	0.579–1	0.659–1
*p* value	<0.001	0.002	<0.001	<0.001	1	<0.001	<0.001	0.928	<0.001
Cut off value	1.45	6.71	6.47	15.2	3.1	2	4.1	0.01	4.34
Sensitivity (%)	78.8	80.0	62.5	87.9	20	75.0	87.9	60	87.5
Specificity (%)	73.2	75.0	100	73.2	100	88.9	87.8	100	77.8
PPV (%)	70.3	80.0	100	72.5	100	85.7	85.3	100	77.8
NPV (%)	81.1	75.0	75.0	88.2	50	80.0	90	66.7	87.5
Cut off value	1.45	6.71	6.47	15.2	3.1	2	4.1	0.01	4.34

## Data Availability

The data presented in this study are available on request from the corresponding author. The data are not publicly available due to ethics.

## Abbreviations

The following abbreviations are used in this manuscript:

sIgE	Specific IgE
sIgG4	Specific IgG4
*D. farinae*	*Dermatophagoides farinae*
ECP	Eosinophilic Cationic Protein
ROC	Receiver operating characteristic
AUC	Area Under the Curve
IL-10	Interleukin-10
OFC	Oral Food Challenge
PPV	Positive Predictive Value
NPV	Negative Predictive Value
